# A Rare Presentation of Primary Hyperparathyroidism with Concurrent Aldosterone-Producing Adrenal Carcinoma

**DOI:** 10.1155/2015/910984

**Published:** 2015-06-16

**Authors:** Mario Molina-Ayala, Claudia Ramírez-Rentería, Analleli Manguilar-León, Pedro Paúl-Gaytán, Aldo Ferreira-Hermosillo

**Affiliations:** ^1^Endocrinology Department, Hospital de Especialidades, Centro Médico Nacional Siglo XXI, IMSS, Cuauhtémoc 330, Colonia Doctores, 06720 Mexico City, DF, Mexico; ^2^Experimental Endocrinology Investigation Unit, Hospital de Especialidades, Centro Médico Nacional Siglo XXI, IMSS, Cuauhtémoc 330, Colonia Doctores, 06720 Mexico City, DF, Mexico

## Abstract

Aldosterone-producing adrenocortical carcinomas are an extremely rare cause of hyperaldosteronism (<1%). Coexistence of different endocrine tumors warrants additional screening for multiple endocrine neoplasia syndromes, especially in young patients with large or malignant masses. We present the case of a 40-year-old man with a history of hypertension that presented with an incidental left adrenal tumor during an ultrasound performed for nephrolithiasis. Biochemical assessment showed a mildly elevated calcium (11.1 mg/dL), high parathyroid hormone, and a plasma aldosterone concentration/plasma renin activity ratio of 124.5 (normal < 30), compatible with primary hyperparathyroidism with a concomitant primary hyperaldosteronism. A Tc99m-MIBI scintigraphy showed an abnormally increased tracer uptake in the right superior parathyroid and abdominal computed tomography confirmed a left adrenal tumor of 20 cm. The patient underwent parathyroidectomy and adrenalectomy with final pathology reports of parathyroid hyperplasia and adrenal carcinoma with biochemical remission of both endocrinopathies. He was started on chemotherapy, but the patient developed a frontal cortex and an arm metastasis and finally died less than one year later.

## 1. Introduction

Primary hyperparathyroidism due to multiple gland hyperplasia is the most common presentation of parathyroid disease in patients with multiple endocrine neoplasia type 1 (MEN1), which also includes pituitary tumors (mostly prolactinomas) and tumors in endocrine pancreas and duodenum. However, other gastroenteropancreatic neuroendocrine tumors, adrenocortical adenomas, or thyroid nodules can occur in MEN1 [1]. Almost 50% of MEN1 patients could develop adrenal adenomas or hyperplasia, which are mainly nonfunctioning and benign [2]. Adrenocortical carcinomas (ACC) are rare manifestations of MEN1 with an incidence of 6% according to Waldmann et al. [2]. In fact, less than 1% of ACC produce aldosterone, cortisol, or androgens. Aldosterone-producing adrenocortical carcinomas (APAC) are infrequent but are highly aggressive and recurrent [3] and could evolve from initially small and clinically nonfunctional adenomas.

The purpose of this paper is to report an unusual association of primary hyperparathyroidism with an APAC that supports the need for extensive endocrinological assessments in patients with adrenal carcinomas and any atypical presentation of neuroendocrine disease.

## 2. Case Report

A 40-year-old man presented with a 4-year history of hypertension treated with aldosterone receptor antagonist (ARA2) and in 2008 a left lithotripsy. He had a family history of type 2 diabetes and cancer (type not specified). He presented with left lumbar pain, dysuria, and hematuria that improved with antibiotic therapy, multiple analgesics, and spontaneous expulsion of a 0.5 × 0.5 cm calculus. During the urologic assessment, the renal ultrasound detected an incidental left adrenal tumor. In the next months, he developed leg cramps and early satiety and lost approximately 8 kg of body weight.

Laboratory examinations reported serum sodium 138 mEq/L (normal 135–145 mEq/L), potassium 4.5 mEq/L (normal 3.5–5 mEq/L), calcium 11.1 mg/dL (normal 8.4–10.2 mg/dL), phosphate 2.5 mg/dL (normal 2.7–4.5 mg/dL), magnesium 1.7 mg/dL (normal 1.6–2.6 mg/dL), and albumin 4.2 g/dL (normal 3.4–4.8 g/dL). PTHi was also elevated (151 pg/mL, normal: 10–65 pg/mL) and vitamin D was normal (30 ng/mL). The screening for adrenal incidentaloma included urinary-free cortisol level of 104.61 mg/24 h (normal < 130 mg/24 h), dehydroepiandrosterone sulfate of 150 *µ*g/dL (normal: 95–530 *µ*g/dL), plasmatic aldosterone concentration (PAC) of 24.9 ng/dL, plasmatic renin activity (PRA) of 0.207 ng/mL/h, PAC/PRA (aldosterone/renin ratio) of 124.5 (normal < 30), urinary normetanephrine of 377 *µ*g/24 h (normal 82–500 *µ*g/24 h), and urinary metanephrine <28 *µ*g/24 h (normal 45–290 *µ*g/24 h).

**Figure 1:**
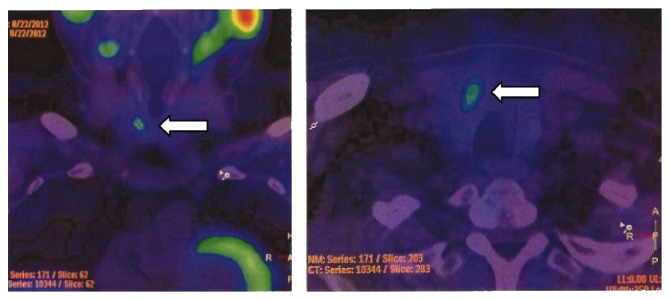
Parathyroid Tc99m-MIB scintigraphy. Images showed an increased uptake of the right superior parathyroid gland on the 150 min delayed image compared with the early 15 min image (white arrow).

**Figure 2:**
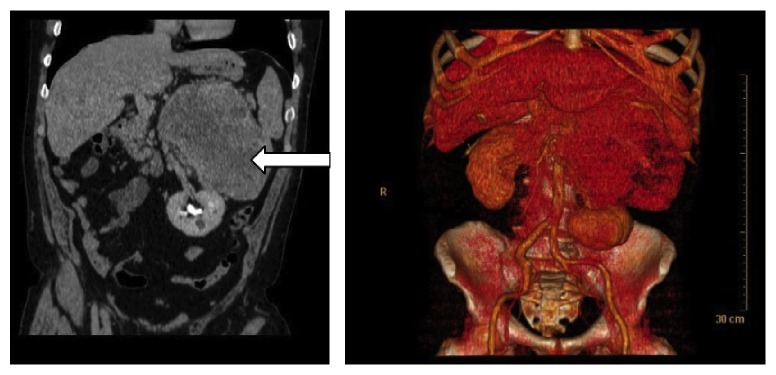
Adrenal CT Image shows a tumor on the left adrenal gland of 202 × 127 × 202 mm (white arrow) that displaces ipsilateral kidney and renal cysts.

After biochemical confirmation of hyperparathyroidism, a Tc99m-MIBI scintigraphy showed an abnormally increased tracer uptake in the right superior parathyroid (Figure 1). The abdominal CT scan confirmed the presence of a giant left adrenal tumor (20 × 13 × 20 cm) (Figure 2). Despite the kidney displacement due to the large tumor, there was no evidence of arterial stenosis. Based on these findings, a diagnosis of primary hyperparathyroidism associated with an aldosterone-producing tumor (highly suspicious of malignancy) was made.

Blood pressure was treated with spironolactone and ARA2. During surgery, enlargement of both left and right parathyroid glands was observed and bilateral parathyroidectomy was successfully done with pathological diagnosis of diffuse hyperplasia of both glands. After surgery, serum calcium decreased to 9 mg/dL, phosphate decreased to 2.7 mg/dL, magnesium decreased to 2 mg/dL, and PTHi decreased to 48.75 pg/mL. Blood pressure was successfully controlled with medical therapy and a left adrenalectomy was performed 5 months later. Histologic study reported a 2750 g tumor with diffuse architecture, areas of necrosis, hemorrhage, and venous invasion, three of the nine criteria previously described by Weiss for malignant adrenal neoplasias which is compatible with the final diagnosis of adrenal adenocarcinoma [4]. Immunohistochemical analysis reported positivity to inhibin alpha and Melan-A (MART-1) and neuron specific enolase and synaptophysin and negativity to PS100 and chromogranin A, which confirmed the diagnosis of a neuroendocrine tumor. Unfortunately, we have no access to the molecular (RT-PCR) or immunohistochemistry techniques that could allow us to identify the type 1 PTH receptor in tissue samples.

**Figure 3:**
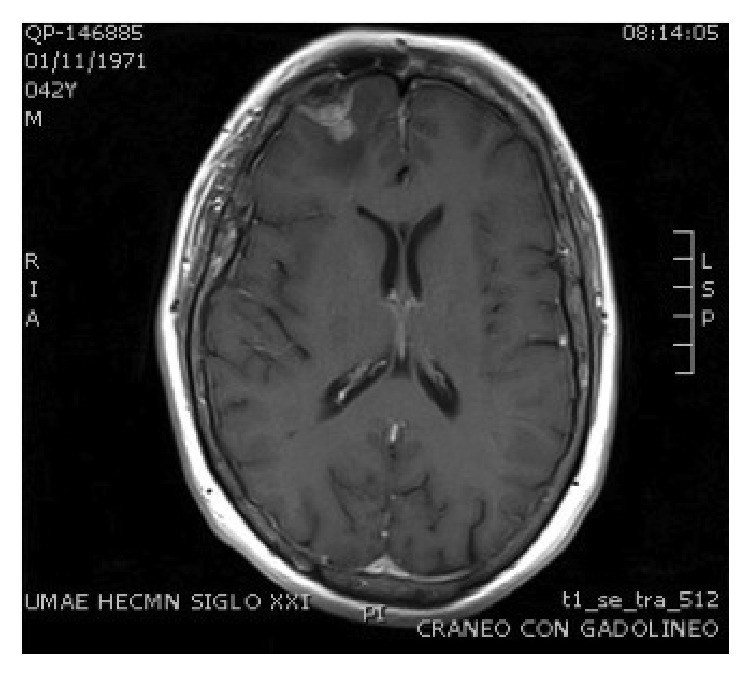
Brain MRI. Image in T1 shows a right frontal tumor of 19 × 21 × 19 mm with associated edema.

After adrenalectomy, spironolactone was discontinued, blood pressure remained under control, and aldosterone levels were normal (6.1 ng/dL, normal < 10 ng/dL). Later, the patient had a generalized seizure and magnetic resonance imaging (MRI) scan showed a tumor in the right frontal area compatible with a metastatic lesion (Figure 3). Afterwards, he presented with severe arm pain that did not respond well to analgesics. A muscular biopsy performed reported carcinoma metastases. Despite chemotherapy treatment, he died 9 months later.

## 3. Discussion

Multiple endocrine neoplasia type 1 is an autosomal dominant disorder characterized by the presence of endocrine tumors in several organs, including parathyroid, gastroenteropancreatic tract, and pituitary [5]. Parathyroid hyperplasia is the most common manifestation and primary hyperparathyroidism and it is usually the heralding feature of this disease, with a penetrance of 80 to 100%. Second in frequency are the tumors of the anterior pituitary that include adenomas producing prolactin, growth hormone, thyroid-stimulant hormone, adrenocorticotropic hormone, and nonfunctioning tumors. Finally, patients may also develop gastroenteropancreatic tumors such as gastrinomas, insulinomas, glucagonomas, somatostatinomas, VIPomas, or GRFomas and nonfunctioning tumors [6]. Other diseases related to MEN1 include the so-called “carcinoid” tumors (currently called neuroendocrine neoplasms), located mainly in foregut, stomach, bronchus, or timus; skin manifestations that include facial angiofibromas, collagenomas, and macules; neuroendocrine tumors and specific nonendocrine tumors [7]. Although previous studies report that 20 to 40% of the patients could develop adrenocortical tumors, only few of them are functional or become functional during followup [8]. Our case is an extremely unusual presentation because the patient developed these two infrequent features: malignancy and functionality, and they presented synchronously in a young patient. When MEN diagnosis is a possibility, clinical suspicion allows for other tumors to be detected early, in mild or subclinical stages, and they usually turn out to be treatable. However, the more the comorbidities are found, the more difficult the treatment is and the worse the prognosis is, such as in this case.

Hypertension is a common disease, especially in endocrine pathologies. Almost 80% of patients with primary hyperparathyroidism are hypertensive [9]. Paradoxically, PTH seems to be involved in vasodilation through endothelial mechanisms and this could decrease rather than increase blood pressure. However, PTH could induce aldosterone secretion in a dose-dependent manner as previously observed in rat and human adrenocortical cells. In fact, previous immunohistochemistry studies revealed the expression of type 1 PTH receptor in aldosterone-producing tumors and in normal tissues that respond to PTH as well as PTH-related peptide (PTH-rP) through activation of adenylatecyclase/cAMP-dependent protein kinase, phospholipase C/protein kinase C, and cAMP-dependent signaling cascades [10]. It seems that aldosterone production is directly enhanced with facilitated calcium entry in adrenal cells stimulated by PTH and indirectly enhanced through stimulation of renin release with a concomitant release of angiotensin II [11].

A growing bulk of evidence suggests that hyperaldosteronism could increase PTH levels. In murine models, aldosterone infusion increased PTH levels through natriuresis and subsequent hypercalciuria that also decreased ionized calcium and released PTH [12]. Rossi et al. found that, in humans, PTH levels were higher in aldosterone-producing adenomas when compared with hyperplasia, suggesting an aldosterone-dose response [13]. Other authors suggest that an increase in PTH levels is related to an increased level of angiotensin II. In fact treatment with captopril (an angiotensin-converting enzyme inhibitor) lowered PTH by 12% from baseline [14]. Finally, Maniero et al. documented the mineralocorticoid receptor (MR) expression in parathyroid adenoma cells using immunocytochemistry and immunoblotting [15] and Brown et al. documented the presence of angiotensin type 1 receptor even in normal parathyroid tissue [14]. These findings suggest that aldosterone and PTH have a reciprocal but not completely understood interaction.

In our institution, we lack a standardized method to assess vitamin D level, which limits the interpretation of results (normal level observed in our case). This must be taken into account because low vitamin D levels are associated with higher plasma renin activity and angiotensin II concentrations that could also increase aldosterone and PTH levels [16]. This relationship has been explored as a possible therapeutic target with conflicting results [17].

Honda et al. reported the case of a 44-year-old woman with confirmed primary hyperparathyroidism, aldosterone-producing adenoma, and breast cancer [18]. They detected a mutation at codon 541 in exon 10 with a loss of heterozygosity (LOH) in each contralateral allele in parathyroid adenoma and breast cancer tissue but not in adrenocortical adenoma tissue. However, other reports found LOH of the 11q13 in aldosterone-producing adrenocortical adenomas, suggesting that patients with primary hyperaldosteronism should be screened for other components of MEN [19]. Unfortunately, in this case, we could not perform an immunoblotting analysis on isolated tissues in search for a possible mutation in MEN gene. Despite the report of concomitant hyperaldosteronism and PHPT, adrenal carcinoma has not been associated with this presentation. In general terms, adrenal carcinomas are rare and aggressive malignancies with an annual incidence of 0.7 to 2 cases per 1 million population per year [20]. They are more common in women with a female to male ratio of 2 : 1 and have a bimodal age distribution with a peak in childhood and a second peak in the fourth and fifth decades [21]. Functional carcinomas are rare and less than 1% of the cases are associated with Conn's syndrome [22]. In fact, Seccia et al. in 2005 reported that since 1955 there were only 60 cases of APAC [3]. They observed that patients with APAC had a peak of incidence of 40 to 49 years of age, were predominantly women, and had a tumor on the right adrenal. Additionally, they described recurrence in 48% of the patients and metastases that involved liver, lungs, abdomen, abdominal lymph nodes, and ipsilateral adrenal site. This case is also particularly atypical because it involves a male with a right adrenal tumor with metastases to the arm and brain.

Surgery is still the first-line treatment [21] whenever it is possible. It is indicated even in patients with advanced disease. Additionally, the most common systemic drugs used include mitotane, cisplatin, and etoposide alone or in combination with other agents. Adjuvant treatment as radiation therapy could be indicated to treat symptomatic metastatic lesions as well as chemoembolization or radiofrequency ablation [21]. In this case, chemotherapy treatment was initiated, but patient was in such an advanced stage of the disease that it was finally unsuccessful.
